# Proteomic analyses identify HK1 and ATP5A to be overexpressed in distant metastases of lung adenocarcinomas compared to matched primary tumors

**DOI:** 10.1038/s41598-023-47767-5

**Published:** 2023-11-28

**Authors:** Helen Pasternack, Mirjam Polzer, Timo Gemoll, Christiane Kümpers, Thorben Sauer, Pamela Lazar-Karsten, Sofie Hinrichs, Sabine Bohnet, Sven Perner, Franz Friedrich Dressler, Jutta Kirfel

**Affiliations:** 1 Institute of Pathology, University Hospital Schleswig-Holstein, Campus Luebeck, Luebeck, Germany; 2 Institute of Legal Medicine, University Hospital Münster, Münster, Germany; 3 Section for Translational Surgical Oncology and Biobanking, Department of Surgery, University Hospital Schleswig-Holstein, Campus Luebeck, Luebeck, Germany; 4 Department of Pulmonology, University Hospital Schleswig-Holstein, Campus Luebeck, Luebeck, Germany; 5 Pathology, Research Center Borstel, Leibniz Lung Center, Borstel, Germany; 6 Institute of Pathology and Hematopathology, Hamburg, Germany; 7 Institute of Pathology, Charité –Universitätsmedizin Berlin, Corporate Member of Freie Universität Berlin, Humboldt-Universität Zu Berlin, and Berlin Institute of Health, Berlin, Germany

**Keywords:** Lung cancer, Tumour biomarkers

## Abstract

Lung cancer is the leading cause of cancer-related deaths worldwide with lung adenocarcinoma (LUAD) being the most common type. Genomic studies of LUAD have advanced our understanding of its tumor biology and accelerated targeted therapy. However, the proteomic characteristics of LUAD are still insufficiently explored. The prognosis for lung cancer patients is still mostly determined by the stage of disease at the time of diagnosis. Focusing on late-stage metastatic LUAD with poor prognosis, we compared the proteomic profiles of primary tumors and matched distant metastases to identify relevant and potentially druggable differences. We performed high-performance liquid chromatography (HPLC) and electrospray ionization tandem mass spectrometry (ESI–MS/MS) on a total of 38 FFPE (formalin‐fixed and paraffin‐embedded) samples. Using differential expression analysis and unsupervised clustering we identified several proteins that were differentially regulated in metastases compared to matched primary tumors. Selected proteins (HK1, ATP5A, SRI and ARHGDIB) were subjected to validation by immunoblotting. Thereby, significant differential expression could be confirmed for HK1 and ATP5A, both upregulated in metastases compared to matched primary tumors. Our findings give a better understanding of tumor progression and metastatic spreads in LUAD but also demonstrate considerable inter-individual heterogeneity on the proteomic level.

## Introduction

Lung cancer is the leading cause of cancer deaths worldwide, with non-small cell lung cancer (NSCLC) as the prevalent form with a poor 5‐year survival rate of less than 15%^1, 2^. NSCLC is subdivided into three major histological types: squamous cell carcinoma, large cell carcinoma, and adenocarcinoma. Lung adenocarcinoma (LUAD) is the predominant histological type of lung cancer and accounts for about 40% of all cases. It is the most common subtype diagnosed in never-smokers^3^.

Recent advancements in high‐throughput molecular biology technologies have deepened our understanding of the pathology underlying NSCLC and highlighted the significant heterogeneity of NSCLC. Especially in LUAD, sequencing of entire cancer genomes has resulted in the identification of recurrent driver alterations in several genes (e.g., *EGFR, BRAF, ALK, RET, ROS1, TP53*) and frequently transformed signaling pathways. Thereby, new molecular subtypes of LUAD were defined and novel targeted treatment options could be developed.

Despite advances in personalized therapies as well as surgery, radiation and chemotherapy, longevity has not increased significantly. Thus, lung cancer patients’ prognosis is still poor and particularly dependent on the stage of disease while first diagnosis. Patients with stage I tumors can expect a 5‐year survival rate of up to 85%, for locally advanced disease, the survival drops to less than 30% and patients diagnosed with distant metastases have a miserable 5‐year survival rate of less than 5%^2^. However, diagnosis at an early stage is only achieved in each third case of lung cancer^4^. Therefore, in our study we focus on distant metastatic stage LUAD.

Among the various potential biomarkers, especially proteins are significant, because they represent the functional gene products and are comparatively stable^5^. They carry out most biological processes and are therefore directly involved in disease progression. However, complex regulatory systems controlling protein expression levels lead to dynamics of the proteome. Proteomics-based analyses, particularly mass spectrometry (MS), include the examination and classification of overall protein signatures in a quantitative manner. Differentially scaled proteomic technologies are applicable in various research settings. They are used to understand mechanisms of pathogenicity, in the analysis of diagnostic biomarkers, in order to detect differential expression patterns reacting to varying signals as well as functional examination of signaling pathways in several diseases. The collection of high-quality fresh tissue for proteomics-based clinical studies is intricate. Therefore, preserved formalin‐fixed and paraffin‐embedded (FFPE) tissues represent a valuable resource for retrospective studies with subsequent proteomic analyses^6, 7^.

We performed proteomic analysis using high-performance liquid chromatography (HPLC) and electrospray ionization tandem mass spectrometry (ESI–MS/MS) on a total of 38 FFPE samples corresponding to 14 patients with advanced LUAD and available tissue of matched distant metastases. Changes at the protein level between primary and metastatic tissue were detected and differentially expressed proteins were identified and validated using immunoblot. So far, proteomic studies in lung cancer mostly focused on early tumor detection and often used blood samples as sample origin (reviewed in^5^ and^8^). Even in tissue-based studies, metastatic samples were usually not included. Currently, there is only one proteomic study focusing on brain metastatic LUAD^9^. To our knowledge, this is thus the first proteomic study on matched pairs of primary and differently localized metastatic LUAD tissues providing a deeper insight into the proteomic changes during metastatic spread of LUAD.

## Results

Our cohort comprised a total of 38 FFPE samples corresponding to 14 patients diagnosed with LUAD and accessible tissue of primary tumors as well as distant metastases (detailed sample information is given in Supplemental Table S1). Due to the limited availability of resected tissue samples especially of metastases we included several samples gained through clinical autopsies. Patient specific characteristics are summarized in Table 1. For each patient comprehensive molecular profiling was performed using fluorescence *in-situ* hybridization (FISH) and massive parallel sequencing (NGS, Table 1). None of the cases showed targetable gene alterations in *EGFR, BRAF, ALK, RET* or *ROS1*. One case was identified to carry an *ERBB2* amplification and three cases showed the common *KRAS* p.G12C mutation. The most frequently mutated genes were *TP53* in 57% and *KRAS* in 43%.

**Table 1:** Patient cohort.

	ID	Sex	Age at diagnosis [years]	Smoking history (pack years)	Pretreatment with chemotherapy	Number of analyzed metastases (localization)	Relevant detected molecular pathological alterations
	1	Male	70	Yes (20py)	No	1 (Stomach)	*TP53* c.743G > A p.R248Q, *MET* amplification
	2	Female	56	Not available	No	2 (Liver, Brain)	*KRAS* c.34G > T p.G12C, *TP53* c.818G > T p.R273L
	3	Male	56	No	Yes	4 (Bone, Adrenal gland, Liver)	*KRAS* c.34G > T p.G12C
	4	Female	53	Yes	Yes	1 (Adrenal gland)	*STK11* c.827G > C p.G276A
	5	Female	55	Yes	Yes	1 (Adrenal gland)	*KRAS* c.35G > C p.G12A, *TP53* c.1009C > T p.R337C, *MET* amplification
	6	Male	48	Yes	No (primary tumor), Yes (metastasis)	1 (Kidney)	*MET* amplification
	7	Male	48	Yes	Yes	3 (Brain, Bone, Adrenal gland)	*TP53* c.487 T > G p.Y163D, *ERBB2* amplification, *MET* amplification
	8	Male	62	Yes (70py)	No	2 (Liver, Small intestine)	*TP53* c.473G > C p.R158P
	9	Male	64	Yes (50py)	not available	3 (Liver, Bone, Kidney)	None
	10	Male	56	Yes	Yes	1 (Liver)	BRAF c.1742A > G p.N581S,
	11	Male	68	Yes (52py)	Yes	1 (Liver)	None
	12	Male	76	Yes (1py)	No	1 (Adrenal gland)	KRAS c.35G > A p.G12A
	13	Male	71	Yes (100py)	No	2 (Liver, Bone)	KRAS c.34G > T p.G12C
	14	Female	60	Yes (30py)	No (primary tumor), Yes (metastasis)	1 (Bone)	KRAS c.34G > T p.G12C
Total:	14					24	

**Figure 1:**
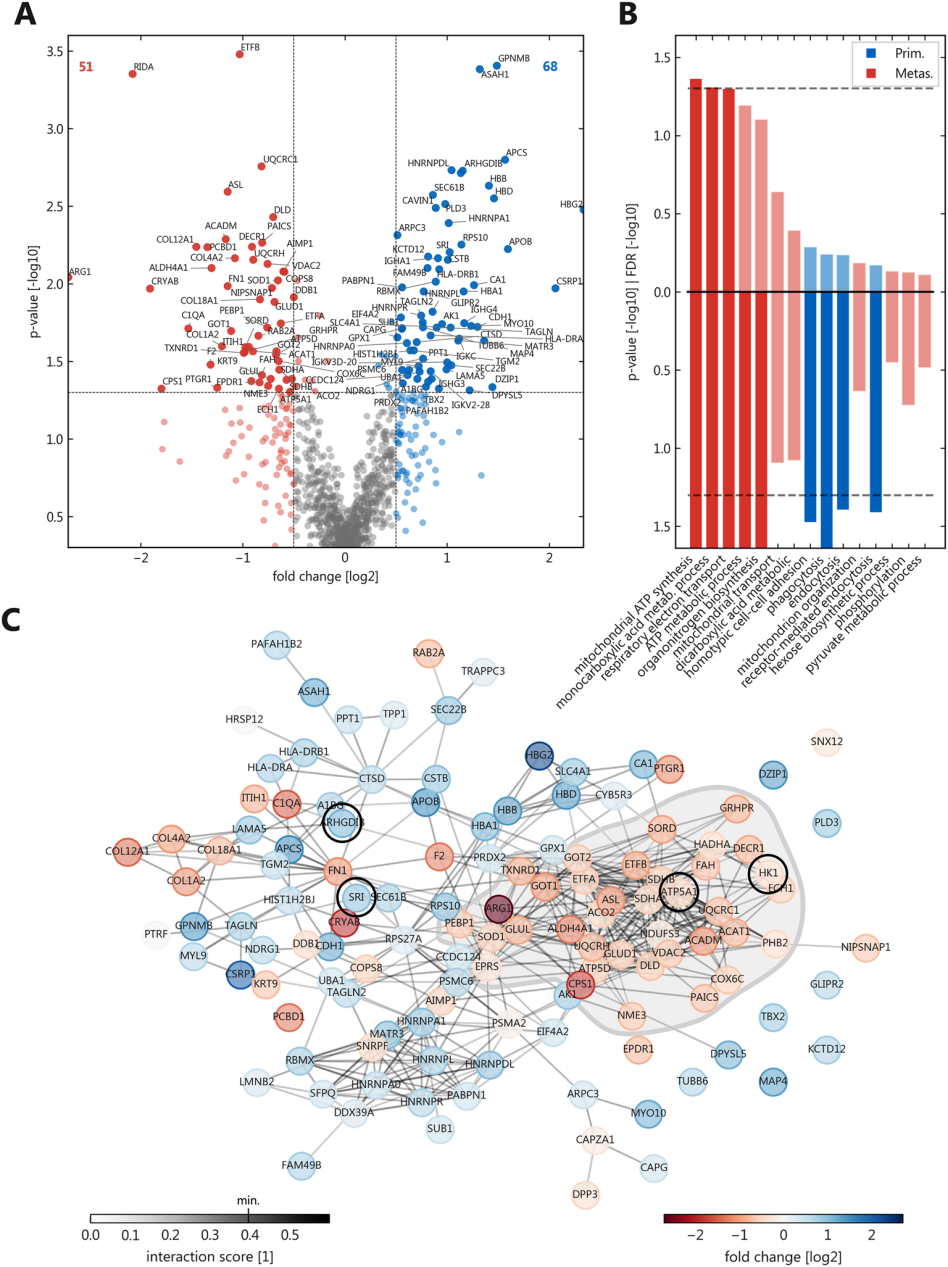
Differential expression between primaries and their metastases. (**A**) Volcano plot with upregulated proteins in metastases on the left side (51, red) and in primaries on the right (68, blue), horizontal line is unadjusted p = 0.05, vertical lines are absolute log_2_ fold changes = 0.5; (**B**) Gene ontology (GO) pathways significantly enriched in primaries or metastases; GO terms ordered by false discovery rate (upward bars) with parallel display of the significance thresholds (0.05; dashed) and unadjusted p-values (downward bars); (**C**) STRING protein–protein interaction network of all significantly regulated proteins from (**A**); negative fold changes represent upregulation in metastases; only connections with more than 0.4 interaction score are shown; light grey visualizes the metastasis-linked cluster of proteins; circled candidate proteins underwent immunoblot validation.

To compare primary tumors and metastases on the proteome level, we performed HPLC and microflow ESI–MS/MS analysis using data-independent acquisition for exact quantification. Spectronaut analysis revealed 1405 distinct proteins identified across all samples (median 1003 per sample). 1055 were identified in ≥ 50% of the samples and were used for subsequent analyses. We first compared the pooled protein expression between primary tumors and metastases (Fig. 1 and Supplemental Table S2). 137 proteins (12.9%) were significantly (unadjusted p ≤ 0.05) differentially expressed between primaries or metastases. Of these 119 had a minimal fold change of 0.5 with overexpression in primaries (68) or metastases (51), respectively (Fig. 1A). The most frequent biological processes belonging to the proteins upregulated in metastases were the oxidation–reduction process, the mitochondrial electron transport, fatty acid beta-oxidation, and angiogenesis. For those upregulated in primaries these were complement activation, receptor-mediated endocytosis, the Fc-gamma receptor signaling pathway involved in phagocytosis, mRNA splicing, and the innate immune response. Of note, a number of metastasis-specific proteins were related to the extracellular matrix/stroma (e.g., the collagen subtypes COL4A2, COL18A1, and COL1A2). To evaluate the functional alterations more comprehensively, we used gene/protein set enrichment analyses (GSEA, Fig. 1B and Supplemental Table S3). Here, significantly enriched pathways were mostly found in the metastasis group (11 out of the top 15 pathways). In line with the biological processes, these were related to cellular energy metabolism, interestingly also mostly involving mitochondrial pathways. For all significantly regulated proteins (unadjusted p ≤ 0.05) a STRING protein–protein interaction network was created (Fig. 1C), which also showed a metastasis-linked cluster of metabolic proteins.

**Figure 2:**
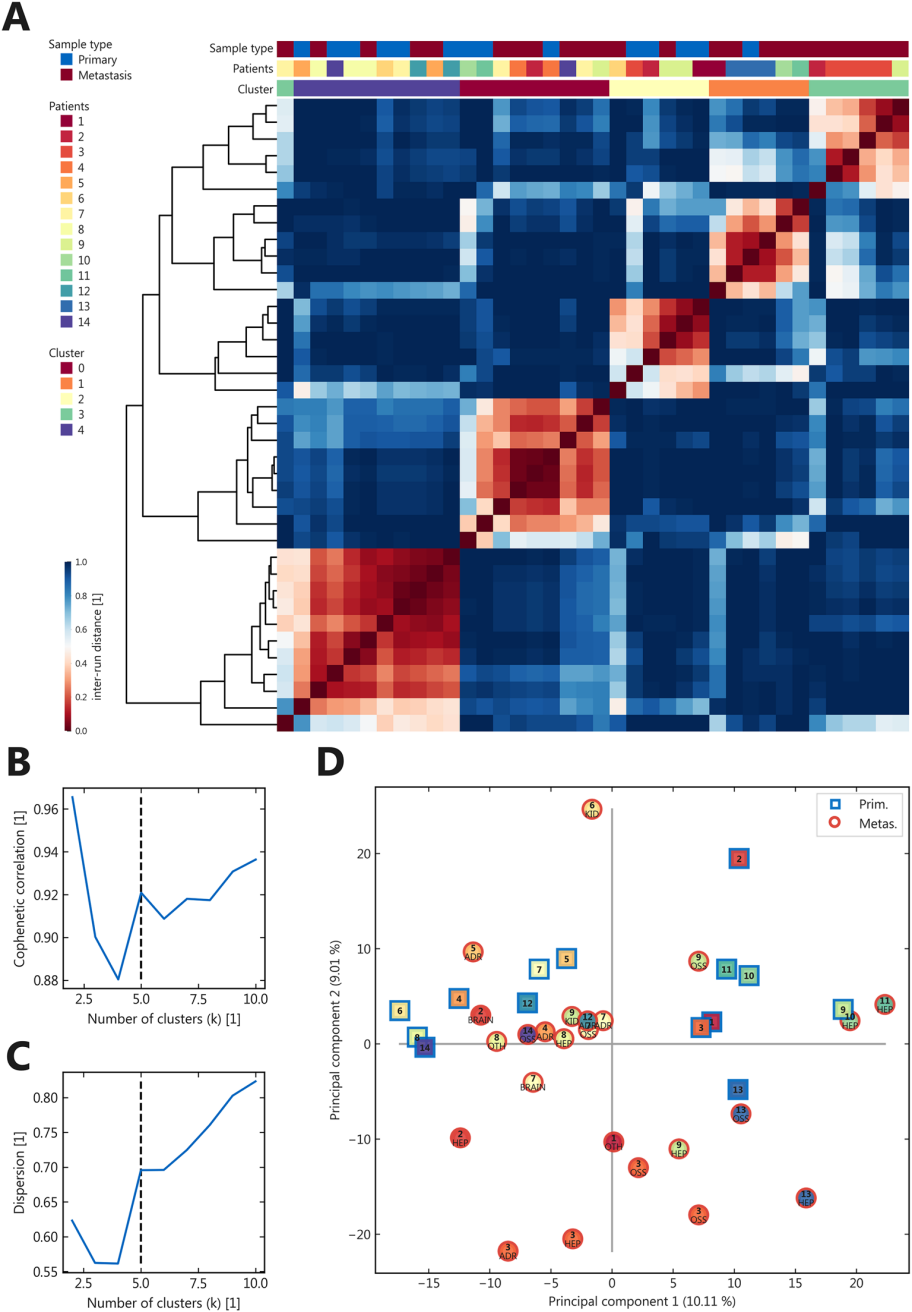
Unsupervised cluster analysis (**A**) Consensus matrix for k = 5 clusters, color indicates stochastic reproducibility across independent runs; (**B**–**C**): Rank determination by cophenetic correlation (**B**) and dispersion (**C**); (**D**) Principal component analysis for the different samples; abbreviated localizations are given for each metastatic sample (*ADR* adrenal gland, *HEP* liver, *KID* kidney, *OSS* bone, *OTH* other).

Due to the high variances in standard differential expression analysis, we used a second, orthogonal, and unsupervised evaluation approach to identify proteomic patterns across primary LUAD samples and metastases. This approach is generally applied to identify patterns across multiple types of quantitative data, including transcript and protein expression data (e.g.^10^). For unsupervised cluster analysis (Fig. 2 and Supplemental Table S4) rank determination by cophenetic correlation and dispersion revealed a distinct local maximum for k = 5 clusters with reasonable cluster separation and stability (Fig. 2A–C). Four of the five identified clusters were composed of a mixture of both primaries and metastatic samples, while one cluster included almost all metastases from one individual patient—highlighting the relevant interindividual heterogeneity. Similarly, a principal component analysis made some separation visible but explained only a minor variance (Fig. 2D). The similarity between matched pairs becomes evident, for example in patients 3, 10, 11, and 13. However, a clustering based on the metastatic locations is not visible. In order to show the effect of imputation, a principle component analysis (PCA) plot of the samples before (100% valid value filter, 334 values) in comparison to the one after imputation (50% valid value filter plus imputation) is given in Supplemental Fig. S1. Omission of imputation leads to less separation by the first two principal components, with a similar sample-wise pattern.

**Figure 3:**
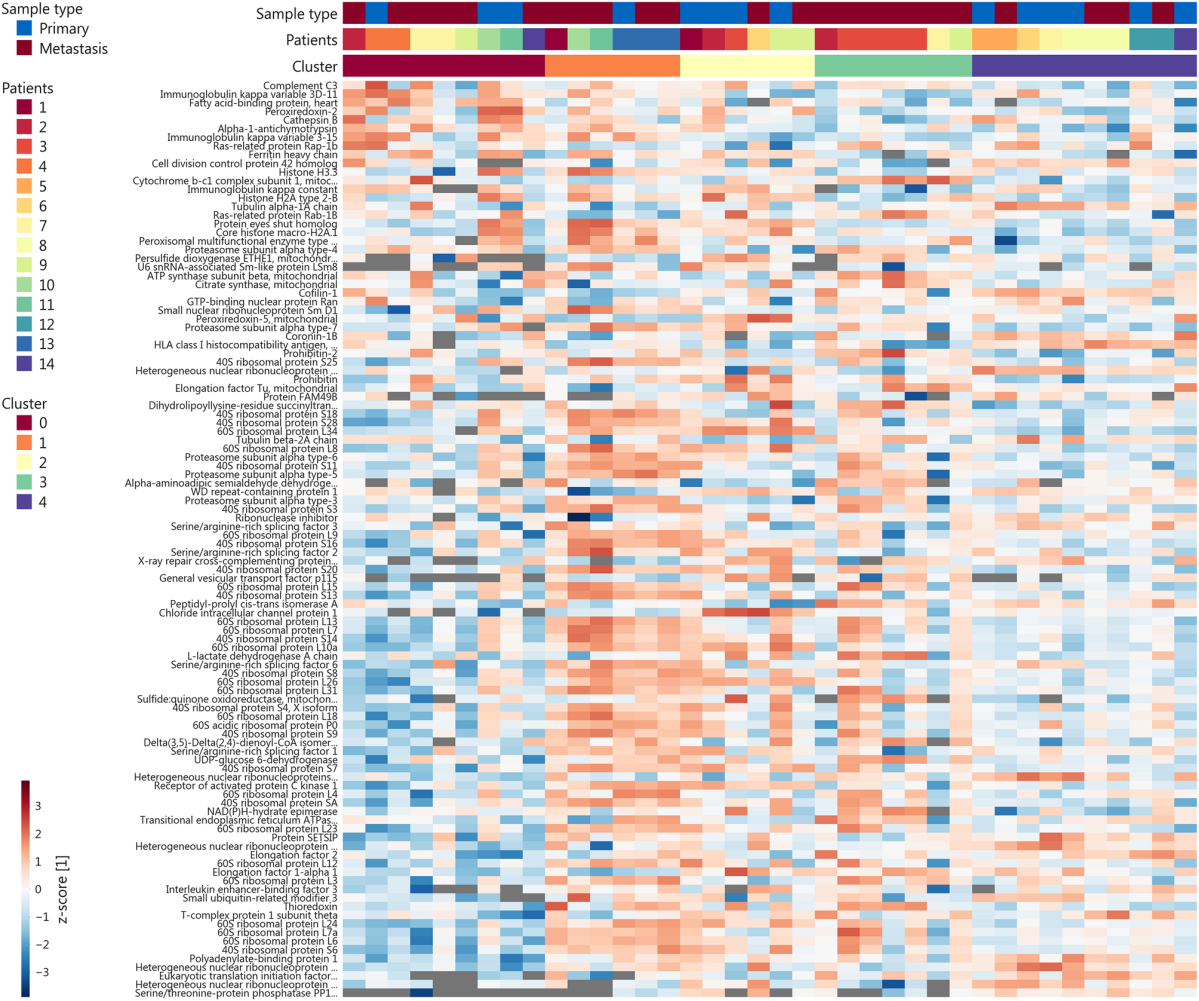
Expression heatmap of cluster-relevant proteins. Log_2_-normalized and zscore-transformed expression data for the 10% most relevant proteins for the clusters from Fig. 2; Missing values in grey.

**Figure 4:**
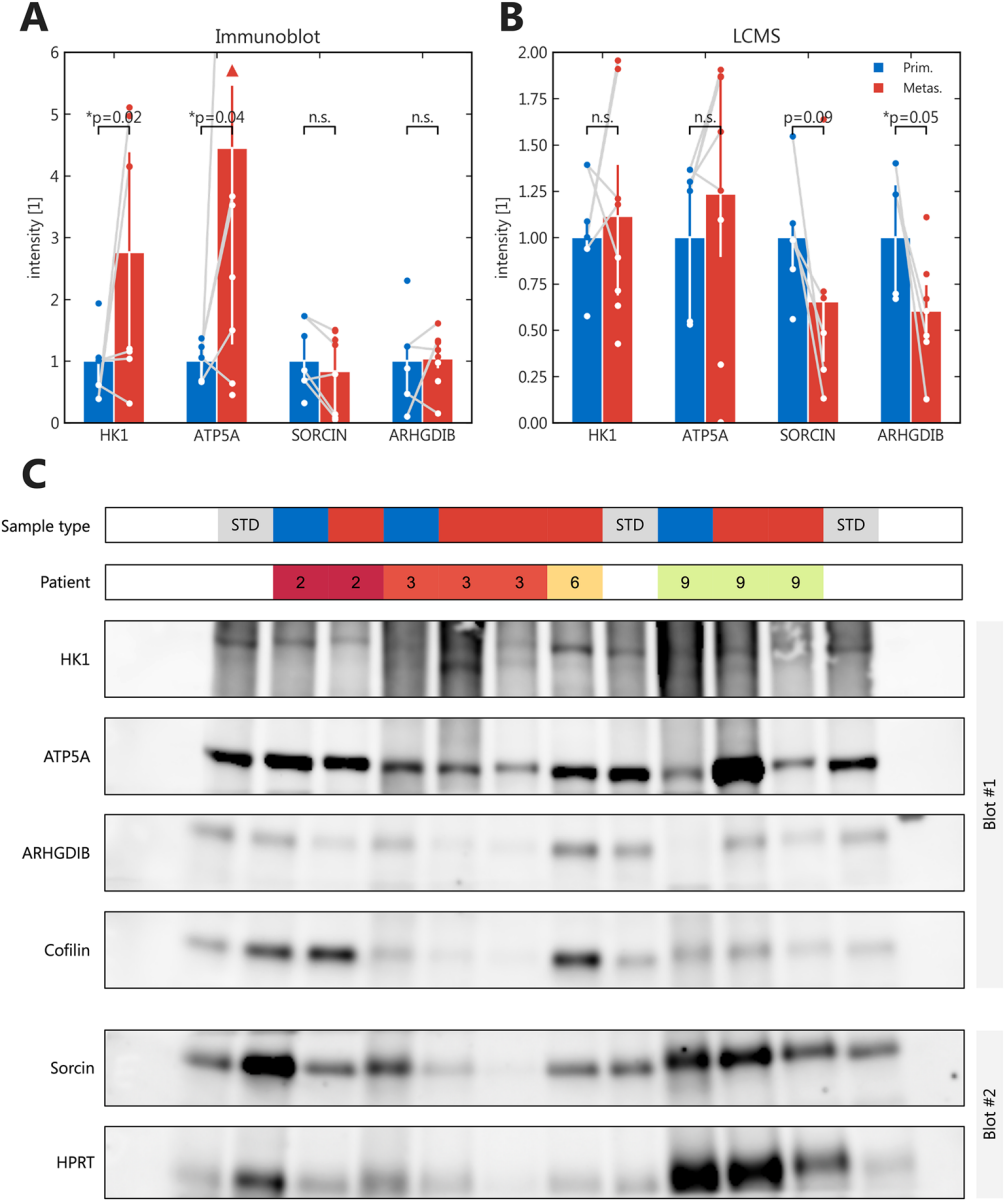
Immunoblot validation. (**A**) Immunoblot results; Normalized densitometric intensities of n = 5 primaries and n = 8 metastases; Whiskers represent interquartile range; p values are from Mann–Whitney-U; light lines link sample pairs; (**B**) Mass spectrometric normalized intensities of the samples from (**A**); (**C**) Exemplary immunoblot; STD = Standards for cross-blot normalization; Sample type blue = Primary; Sample type red = Metastasis; Full-width blots cropped for the specific protein bands; Blot #2 for the quantification of Sorcin with a separate loading control, which matches its molecular weight; complete original blots are presented as Supplemental Figs. S2–S7 with a corresponding sample matrix as Supplemental Table S5; quantitatively compared blots were generated during the same experiment and processed in parallel.

Figure 3 visualizes the 10% most cluster-relevant proteins (protein score > 90th percentile)^11^. The overlap between these 106 cluster-relevant proteins and those differentially expressed in pooled comparison comprised nine proteins (ARHGDIB, HNRNPA1, SRI, CYRIB/FAM49B, HNRNPL, HK1, IGKC, PAFAH1B2 and ATP5A1) and was used to choose proteins likely involved in metastasis for further validation. Due to their potential role in tumorigenesis HK1 and ATP5A (upregulated in metastases) as well as SRI and ARHGDIB (upregulated in primaries) were selected. Quantitative expression was measured in n = 6 primaries, n = 8 matched metastases and n = 2 additional metastatic samples using immunoblotting (Fig. 4). Significant differential expression (p < 0.05) could be confirmed for HK1 and ATP5A, both upregulated in metastases compared to matched primary tumors in immunoblot as well as LC–MS/MS analyses (Fig. 4A, B). An exemplary immunoblot reflecting differential expression is shown in Fig. 4C. All immunoblots are provided as original TIFF files in Supplemental Figs. S2–S7 with a corresponding sample matrix given as Supplemental Table S5. SRI and ARHGDIB did not show significant differences in the immunoblot analysis.

In total, our analyses identified several metabolic proteins with differential expression between primary LUAD and matched distant metastases. HK1 and ATP5A could be validated. However, we also observed considerable inter-individual heterogeneity.

## Discussion

Lung cancer is the leading cause of cancer-related mortality worldwide and lung adenocarcinoma (LUAD) is the most common form of lung cancer with a poor 5-year survival rate of less than 15%^1^. Prognosis for lung cancer patients strongly depends on the stage of disease at time of diagnosis and the presence of metastasis is the major factor for low survival rates^2^. Therefore, there is an urgent need to discover processes and signaling pathways involved in metastasis formation in LUAD. In our study we compared the proteomic profiles measured by high-performance liquid chromatography (HPLC) and electrospray ionization tandem mass spectrometry (ESI–MS/MS) of primary LUAD samples to those of matched distant metastases.

Our cohort comprised a total of 38 FFPE samples corresponding to 14 patients diagnosed with LUAD and accessible tissue of primary tumors as well as distant metastases. The most frequently mutated genes in our cohort were *TP53* in 50% and *KRAS* in 29%, reflecting a typical distribution in a LUAD cohort. *KRAS* mutation is known to be the most common gain-of-function alteration, accounting for around 30% of LUADS in western countries^12^.

In recent years, proteomic studies have become a widely used research tool in analyzing cancer biology, complementing the results of genetic profiling. As most biological functions are carried out by proteins, protein profiles can often represent even more accurately a disease state and thus be a more reliable and quantitative tool to discover new cancer biomarkers. Mass spectrometry (MS) techniques allow the identification of differentially expressed proteins in small quantities of tumor samples^13, 14^. As fresh frozen tissue with corresponding clinical data is often not available for retrospective analyses, several studies showed the feasibility of using stored FFPE tissues for MS-based comprehensive proteomic profiling^6, 7^. So far, most proteomic studies on lung cancer focused on the differentiation of histological subtypes or early diagnosis of malignant disease^15–21^. A very recent study analyzed also distant metastatic tissue, but included only brain metastases^9^. To our knowledge, our study is now the first proteomic study on matched pairs of primary and differently located metastatic LUAD tissues providing a deeper insight into the proteomic changes during the metastatic spread of LUAD.

We identified 1405 proteins across all samples with 1055 shared by at least 50% of the samples. Our differential expression analysis between primary tumors and their corresponding metastases revealed 137 proteins significantly upregulated in primaries or metastases respectively. Another recent LC–MS-based proteomic study on 22 LUAD patients using fresh frozen tissue samples revealed 365 and 366 proteins differentially expressed in early-stage (I-II) or advanced-stage (III-IV) LUAD compared to normal tissue, respectively^22^. Comparable to our study, the authors identified 155 proteins dysregulated between early- and advanced-stage tumors. Their PCA showed a clear separation between four clusters corresponding to different stages and normal vs. tumor tissue. As in our cluster analysis as well as PCA the similarity between matched pairs of the same patient becomes evident and emphasizes the importance of using matched tissue samples for comparative analysis, as we did in our study.

Recently, Gillette et al. published a comprehensive proteogenomic characterization of 110 LUAD and 101 matched normal adjacent tissues (cryopulverized tissue). They revealed four subgroups defined by key driver mutations, country, and gender and identified new therapeutic targets. The study, however, did not include stage IV cancers with distant metastases. It is thus not surprising, that there is no overlap with the herein identified candidate proteins^23^. Another large deep-scale proteogenomics study of LUAD in Taiwanese population^24^ and a comprehensive proteogenomics analysis of 103 LUAD in chinese patients^25^ were published in recent years.

There are several proteomic studies on LUAD tumor progression that compare different stages of the disease. Kawamura et al. identified 81 proteins significantly differentially expressed in stage IA compared to IIIA LUAD^26^. Further analysis revealed NAPSA to be significantly reduced expressed in advanced stage tumors as well as hAG-2 highly expressed in stage IIIA vs. IA LUAD. Additionally, differential expression of hAG-2 was related to regional lymph node metastasis^27^. Also, the study of Hsu et al. focused on lymph node metastasis in LUAD^28^. They identified 133 differentially expressed proteins and selected six of them for further validation (ERO1L, PABPC4, RCC1, RPS25, NARS, and TARS). All of these studies were based on non-metastatic cases and further work identifying biomarkers for distant metastasis formation in LUAD is still lacking. Therefore, our study included only cases with distant metastasis and no early-stage tumors.

A recent study by Woldmar et al.^9^ conducted proteomic profiling on 20 surgically resected primary and brain metastatic LUAD samples. They identified 1496 proteins differentially expressed between primary tumors and corresponding metastases. Pathways activated in primary tumors were associated with the immune system, cell–cell/matrix interactions and migration, whereas metastatic tumor samples displayed overrepresentation of pathways related to metabolism, translation or vesicle formation. In part, these results correspond to the pathways connected with differentially expressed proteins we detected in our study. Similar to Woldmar et al. we found distant metastases to be for example associated with metabolic processes, whereas primary tumors showed amongst others overrepresentation of pathways related to the immune system. However, several particular pathways as well as individual biomarker candidates identified in the different studies do not correspond. This might be due to the fact that instead of analyzing only brain metastases we included also distant metastases of other locations.

Using gene/protein set enrichment analyses we mostly detected significantly enriched pathways in the metastasis group (11 out of the top 15 pathways). In line with the biological processes associated with differentially expressed proteins, these were related to cellular energy metabolism, especially involving mitochondrial pathways. The importance of mitochondrial processes for lung cancer initiation and progression is also described in other studies (e.g.^29^ or reviewed in^30^). Of note, Chuang et al. discovered a specifically altered mitochondrial functionality related to the metastatic cell state of LUAD and that this association could also be used therapeutically^31^.

In our study, the overlap between the 137 differentially expressed proteins and 106 most relevant proteins identified by cluster analysis revealed 9 candidate proteins involved in metastasis formation of LUAD. Of these, four were chosen for validation by immunoblotting: Hexokinase 1 and ATP Synthase F1 Subunit Alpha (HK1, ATP5A, upregulated in metastases) as well as Sorcin and RhoGDP Dissociation Inhibitor Beta (SRI, ARHGDIB, upregulated in primaries). All four candidates have previously been reported to be likely involved in tumorigenesis and partially even in lung cancer. For example, overexpression and amplification of the calcium-binding protein Sorcin has been described for different cancer entities, including lung cancer^32^. Additionally, the association between SRI overexpression and resistance to gemcitabine could repeatedly be shown. Qu et al. identified 14 proteins related to gemcitabine resistance in NSCLC cell lines, among them SRI^33^, which has previously been found to be overexpressed in several multidrug-resistant cell lines^34^. Also, ARHGDIB is reported to be involved in lung cancer tumorigenesis^35^. It was initially shown to be a metastasis suppressor in bladder cancer and later found to be lost in many metastatic tumors^36^.

In our validation, significant differential expression could be confirmed for HK1 and ATP5A, both upregulated in metastases compared to matched primary tumors in immunoblot and LC–MS/MS analyses. ATP5A itself has not yet been described to be associated with lung cancer, but another ATP synthase subunit could already be identified as biomarker for LUAD by Chen and colleagues^37^. They identified nine enzymatic proteins significantly overexpressed in LUAD compared to adjacent normal lung tissue using 2DGE and MALDI-MS or peptide sequencing, including the ATP synthase subunit D (ATP5D). Additionally, it has been reported that inhibiting the ATP synthase suppresses proliferation and growth of lung cancer cells^38^. ATP5A is furthermore described as shared drug target for aging and dementia^39^. The hexokinase HK1 is involved in glycolysis (and in part bound to the mitochondrial outer membrane). Its herein observed differential expression thus corresponds to the detected metastasis-linked cluster of metabolic proteins, mostly involving mitochondrial pathways. We found HK1 to be overexpressed in metastases compared to primary tumors. So far, HK1 was rather described to be expressed in normal tissues, whereas cancer cells often show additional or alternative expression of the HK2 isoform^40, 41^. HK2 was detected to be required for tumor initiation and maintenance in mouse models of KRAS-driven lung cancer^40^ and HK1 knock-out lung cancer cells expressing only HK2 were shown to be sensitive to HK2 silencing-induced cytostasis^41^. In hepatocellular cells HK1 expression correlates with resistance to tyrosine kinase inhibition and its function could be impaired by Lonidamine, a glycolysis inhibitor that inhibits the activity of mitochondrially bound hexokinases^42, 43^. In order to exclude that differential expression of HK1 and ATP5A is caused by an underlying tissue-specific expression we checked protein expression using the human protein atlas^44^. Both proteins are described to be expressed ubiquitously in a non-tissue-specific manner, especially without enhanced expression in any of the herein analyzed localizations. Our validation cohort comprised samples from the discovery cohort. Therefore, an additional validation on a larger and independent cohort would be desirable in the future.

We observed heterogeneous protein expression profiles of matched primary tumors and their distant metastases across patients. Nonetheless, several mostly metabolic proteins were associated with the metastatic state. HK1 and ATP5A could be identified and validated as candidate proteins. These findings give a better understanding of tumor progression and metastasis formation and might help to improve biomarker-based diagnosis and prognosis prediction.

## Methods

### Study design and sample selection

This study has been granted approval by the ethics committee of the University Luebeck (project code AZ 16-277, AZ 16-278). The ethics committee assesses the appropriateness of the design of the retrospective study, in which the samples were included completely anonymized. The requirement for obtaining informed consent has been waived. All investigations were carried out in adherence to the principles in the Declaration of Helsinki.

In total, 38 samples corresponding to 14 patients with advanced lung adenocarcinoma and available tissue of matched distant metastases were identified. Of these, primary tumor tissue from 9 patients and metastases tissue from 12 patients were harvested in clinical autopsies. Patients were annotated by sex, age at diagnosis and smoking status. Detailed information on pretreatment with chemotherapy, localizations, and number of analyzed metastases for each patient is shown in Table 1.

### Histological and molecular pathological characterization

Histological analyses on formalin-fixed/paraffin-embedded (FFPE) tumor blocks were performed in the Institute of Pathology of the University Hospital Schleswig–Holstein, Campus Luebeck. Histology of each case including growth pattern was assessed by senior pathologists experienced in lung pathology. Using H&E-stained slides, tumor areas were marked and tumor cell content was estimated.

For each case, tissue areas with preferably high tumor cell content (mean: 69%, standard deviation: 19%, CV: 0.27, Supplemental Table S1) were selected for nucleic acid extractions. Isolation of genomic DNA was performed using the Maxwell RSC DNA FFPE Kit and the Maxwell RSC instrument (Promega, Fitchburg WI, U.S.A.). DNA samples were quantified using the Qubit fluorimeter (TermoFisher, Waltham MA, U.S.A.). To identify genetic alterations in *AKT1, ALK*^*MUT*^*, BRAF, CTNNB1, DDR2, EGFR, ERBB2*^*Mut*^*, ERBB4, FBXW7, FGFR1, FGFR2, FGFR3, KRAS, MAP2K1, MET*^*Mut*^*, NRAS, NOTCH1, PIK3CA, PTEN, STK11, SMAD4* and *TP53* massive parallel sequencing using the Ion AmpliSeq Colon and Lung Cancer Research Panel v2 and Ion PGM sequencing platform (ThermoFisher Scientific) were used. Additionally, the possible presence of *ALK, RET* or *ROS1* translocations as well as amplifications of *MET* and *ERBB2* in the primary tumors was investigated by fluorescence in-situ hybridization (FISH) using the corresponding ZytoLight probes (ALK Z-2124, RET Z-2148, ROS1 Z-2144, MET Z-2087, ERBB2 Z-2017, ZytoVision, Bremerhaven, Germany).

### Protein extraction

For each primary tumor or metastasis tissue areas with preferably high tumor cell content were selected for proteomic analysis and 45 µm sections were cut off and stored at room temperature. To solubilize the proteins 1 ml Heptane was added to each sample, vortexed for 10 s. After 1.5 h at room temperature, 50 µl Methanol were added and the samples were vortexed again. The samples were centrifuged for 2 min at 9000×*g* at room temperature, the supernatant was removed, and the samples dried out for 5 min at room temperature. The QProteome® FFPE Tissue Kit (Qiagen, USA) was used for protein extraction. Subsequently, total protein concentration was determined in triplets using the fluorescence-based EZQ™ Protein Quantification Kit (Life Technologies, USA). Fluorescence visualization was carried out with the Typhoon™ FLA 9000 laser scanner (GE Healthcare). Densitometric analysis was performed using the ImageQuant™ TL software (GE Healthcare).

For each sample 100 µl lysate containing 25 µg protein were purified using methanol and chloroform. The protein pellet was washed with ethanol and dissolved in 1% RapiGest (Waters, USA) in 25 mM Ammonium bicarbonate (ABC) buffer. Proteins were reduced with 50 mM Dithiothreitol (DTT) and incubated at 37 °C at 950 rpm for 1 h. Afterwards, 100 mM iodoacetamide (in ABC buffer) was used to alkylate the proteins by shaking the samples at 37 °C with 950 rpm for 1 h. Proteins were digested using 25 ng/µl Trypsin (Sigma-Aldrich, USA) in ABC buffer over night at 37 °C. Trifluoroacetic acid (5%) was added and the samples were incubated at 950 rpm at 37 °C. The samples were centrifuged and the supernatant was transferred into a new tube, dried out by vacuum centrifugation for 3 days and stored at − 80 °C until further analysis.

### Proteomic analysis by high-performance liquid chromatography (HPLC) and electrospray ionization tandem mass spectrometry (ESI–MS/MS)

With minor adjustments, proteomic analysis was performed as described previously^45^. The samples were solubilized in 2% acetonitrile/0.5% formic acid. Luna C18 (2) (5 μm, 20 × 0.3 cm; Phenomenex, USA) was used as trap column and the samples were desalted for 5 min. An analytical column (LC Column, 3 μm C18 (2), 150 mm × 0.3 mm, Phenomenex, USA) was used to separate the peptides. Analyzation with mass spectrometer and following SWATH (sequential window acquisition of all theoretical mass spectra) were performed according to Sauer et. al.^45^. Thereby, the collision energy (CE) was set to 10 and the updated SWATH Variable Window Calculator V2.0 was used to define the precursor isolation windows.

The mass spectrometry proteomics data have been deposited to the ProteomeXchange Consortium via the PRIDE^46^ partner repository with the dataset identifier PXD042604. Corresponding raw file names can be obtained from Supplemental Table S1.

### SWATH data processing

The software tool Spectronaut v13.2 (Biognosys, Switzerland) was used for the SWATH data processing. First a hybrid spectral library was established from all 38 SWATH runs and five pooled DDA runs using Spectronaut with default settings. The hybrid spectral library was subsequently searched using the default settings with Spectronauts pulsar search engine. The false discovery rate (FDR) was set to 1% at the peptide precursor level and protein level, respectively. Additionally, all proteins considered in this study were identified by at least two peptides. The human UniProtKB/Swiss-Prot database^47^ was used for protein inference from identified peptides.

### Immunoblot

Immunoblotting was performed as described previously^6^. The primary antibodies were anti-HK1 (1:500; monoclonal mouse IgG; antibodies- online ABIN933202, Aachen, Germany), anti-ATP5A (1:1000; monoclonal rabbit IgG; abcam ab176569, Cambridge, UK), anti-Sorcin A (1:1000; polyclonal rabbit IgG, antibodies-online ABIN5014335, Aachen, Germany), anti-ARHGDIB (1:500;polyclonal rabbit IgG, antibodies-online ABIN2855594, Aachen, Germany) as well as loading controls anti-Cofilin (1:1000; Cell Signaling Technology 5175S, Danvers, USA) and anti-HPRT (1:100; Santa-Cruz sc-376938, Dallas, USA). Secondary antibodies were 1:2500 goat anti-rabbit IgG (ThermoFisher 31460, Schwerte, Germany) and 1:2500 goat anti-mouse IgG (ThermoFisher 31430, Schwerte, Germany).

Conditions for relative protein quantitation were ensured^48^ and the linear ranges determined beforehand. Sample-specific protein abundances were normalized to the mean of the same-gel standards prior to normalization to loading controls.

### Bioinformatics and statistical analyses

Data processing and statistical analyses were performed in Python (2.7.17 and 3.9.9) using the modules nimfa 1.4.0, gseapy 0.10.8 (permutation_type = 'phenotype', permutation_num = 100, method = 't_test', processes = 4, seed = 7), matplotlib 2.2.5, numpy 1.16.1, sklearn 0.20.4 (including decomposition.PCA with default settings), pandas 0.24.2, scipy 1.2.2, and seaborn 0.9.1. The raw data was filtered for proteins quantified in at least 50% of all samples. Data was normalized using Normics_median_^49^ based on the top 100 invariant proteins. Significance for differential expression was calculated with Mann–Whitney-U tests (unadjusted due to comparison to orthogonal unsupervised evaluation). Due to the unequal number of metastases per primary, a more conservative unpaired statistical approach was chosen over paired statistical tests to avoid biased weights across samples. Additionally, Benjamini–Hochberg adjusted p-values are included as an additional worksheet (“adjusted”) in Supplemental Table S2. Unsupervised non-negative matrix factorization was performed on all proteins for k = 2 up till k = 10, with missing values replaced by the mean of all valid values. The mean was chosen over minimum/low values or other more sophisticated methods as a conservative approach (to reduce power rather than introducing biases) in this setting of relatively high missingness (at random) and known performance heterogeneity in FFPE samples in line with suggestions from the literature^50^. Overall, missing values were not imputed for any test, except for PCA and unsupervised cluster analysis. The local maximum at k = 5 was chosen as it demonstrated a distinctive peak for both cophenetic correlation and dispersion. Relevance scores were computed as implemented in the nimfa package^51^ defined by Kim and Park^11^. For gene set enrichment analyses (GSEA) the 2018 gene ontology terms for biological processes were used. The STRING network was created on string-db.org^52^.

### Supplementary Information

Supplementary Figure S1.

Supplementary Figure S2.

Supplementary Figure S3.

Supplementary Figure S4.

Supplementary Figure S5.

Supplementary Figure S6.

Supplementary Figure S7.

Supplementary Table S1.

Supplementary Table S2.

Supplementary Table S3.

Supplementary Table S4.

Supplementary Table S5.

## Data Availability

The mass spectrometry proteomics data have been deposited to the ProteomeXchange Consortium via the PRIDE^46^ partner repository with the dataset identifier PXD042604.

## References

[1] Bray F (2018). Global cancer statistics 2018: GLOBOCAN estimates of incidence and mortality worldwide for 36 cancers in 185 countries. CA.

[2] Myers, D. J. & Wallen, J. M. *Lung Adenocarcinoma* (*StatPearls*, 2022).

[3] Subramanian J, Govindan R (2007). Lung cancer in never smokers: A review. J. Clin. Oncol..

[4] Little AG, Gay EG, Gaspar LE, Stewart AK (2007). National survey of non-small cell lung cancer in the United States: Epidemiology, pathology and patterns of care. Lung Cancer.

[5] Gasparri R, Sedda G, Noberini R, Bonaldi T, Spaggiari L (2020). Clinical application of mass spectrometry-based proteomics in lung cancer early diagnosis. Proteomics.

[6] Dressler FF (2022). Systematic evaluation and optimization of protein extraction parameters in diagnostic FFPE specimens. Clin. Proteom..

[7] Friedrich C (2021). Comprehensive micro-scaled proteome and phosphoproteome characterization of archived retrospective cancer repositories. Nat. Commun..

[8] Baran K, Brzezianska-Lasota E (2021). Proteomic biomarkers of non-small cell lung cancer patients. Adv. Respir. Med..

[9] Woldmar N (2023). Proteomic analysis of brain metastatic lung adenocarcinoma reveals intertumoral heterogeneity and specific alterations associated with the timing of brain metastases. ESMO Open.

[10] Robertson AG (2017). Comprehensive molecular characterization of muscle-invasive bladder cancer. Cell.

[11] Kim H, Park H (2007). Sparse non-negative matrix factorizations via alternating non-negativity-constrained least squares for microarray data analysis. Bioinformatics.

[12] Ghimessy A (2020). Current therapy of KRAS-mutant lung cancer. Cancer Metastasis Rev..

[13] Indovina P (2013). Mass spectrometry-based proteomics: The road to lung cancer biomarker discovery. Mass Spectrom. Rev..

[14] Wang H (2016). The clinical impact of recent advances in LC-MS for cancer biomarker discovery and verification. Expert Rev. Proteomics.

[15] Li LS (2004). Proteomic analysis distinguishes basaloid carcinoma as a distinct subtype of nonsmall cell lung carcinoma. Proteomics.

[16] Rho JH, Roehrl MH, Wang JY (2009). Tissue proteomics reveals differential and compartment-specific expression of the homologs transgelin and transgelin-2 in lung adenocarcinoma and its stroma. J. Proteome Res..

[17] Zeng GQ (2012). Identification of candidate biomarkers for early detection of human lung squamous cell cancer by quantitative proteomics. Mol. Cell. Proteom..

[18] Rodriguez-Pineiro AM, Blanco-Prieto S, Sanchez-Otero N, Rodriguez-Berrocal FJ, de la Cadena MP (2010). On the identification of biomarkers for non-small cell lung cancer in serum and pleural effusion. J. Proteomics.

[19] Li Y (2013). Aberrant Mucin5B expression in lung adenocarcinomas detected by iTRAQ labeling quantitative proteomics and immunohistochemistry. Clin. Proteomics.

[20] Chang YK (2016). Haptoglobin is a serological biomarker for adenocarcinoma lung cancer by using the ProteomeLab PF2D combined with mass spectrometry. Am. J. Cancer Res..

[21] Ciereszko A (2019). Identification of protein changes in the blood plasma of lung cancer patients subjected to chemotherapy using a 2D-DIGE approach. PLoS ONE.

[22] Kelemen O (2020). Proteomic analysis enables distinction of early- versus advanced-stage lung adenocarcinomas. Clin. Transl. Med..

[23] Gillette MA (2020). Proteogenomic characterization reveals therapeutic vulnerabilities in lung adenocarcinoma. Cell.

[24] Chen YJ (2020). Proteogenomics of non-smoking lung cancer in east Asia delineates molecular signatures of pathogenesis and progression. Cell.

[25] Xu JY (2020). Integrative proteomic characterization of human lung adenocarcinoma. Cell.

[26] Kawamura T (2010). Proteomic analysis of laser-microdissected paraffin-embedded tissues: (1) Stage-related protein candidates upon non-metastatic lung adenocarcinoma. J. Proteomics.

[27] Nishimura T (2010). Proteomic analysis of laser-microdissected paraffin-embedded tissues: (2) MRM assay for stage-related proteins upon non-metastatic lung adenocarcinoma. J. Proteomics.

[28] Hsu CH (2016). Identification and characterization of potential biomarkers by quantitative tissue proteomics of primary lung adenocarcinoma. Mol. Cell. Proteomics.

[29] Han M (2023). Spatial mapping of mitochondrial networks and bioenergetics in lung cancer. Nature.

[30] Roberts ER, Thomas KJ (2013). The role of mitochondria in the development and progression of lung cancer. Comput. Struct. Biotechnol. J..

[31] Chuang C-H (2021). Altered mitochondria functionality defines a metastatic cell state in lung cancer and creates an exploitable vulnerability. Cancer Res..

[32] Shabnam B (2018). Sorcin a potential molecular target for cancer therapy. Transl. Oncol..

[33] Qu Y, Yang Y, Liu B, Xiao W (2010). Comparative proteomic profiling identified sorcin being associated with gemcitabine resistance in non-small cell lung cancer. Med. Oncol..

[34] Qi J (2006). Overexpression of sorcin in multidrug resistant human leukemia cells and its role in regulating cell apoptosis. Biochem. Biophys. Res. Commun..

[35] Niu H, Li H, Xu C, He P (2010). Expression profile of RhoGDI2 in lung cancers and role of RhoGDI2 in lung cancer metastasis. Oncol. Rep..

[36] Gildea JJ (2002). RhoGDI2 is an invasion and metastasis suppressor gene in human cancer. Cancer Res..

[37] Chen G (2002). Proteomic analysis of lung adenocarcinoma: Identification of a highly expressed set of proteins in tumors. Clin. Cancer Res..

[38] Chang HY (2012). Ectopic ATP synthase blockade suppresses lung adenocarcinoma growth by activating the unfolded protein response. Cancer Res..

[39] Goldberg J (2018). The mitochondrial ATP synthase is a shared drug target for aging and dementia. Aging Cell.

[40] Patra KC (2013). Hexokinase 2 is required for tumor initiation and maintenance and its systemic deletion is therapeutic in mouse models of cancer. Cancer Cell.

[41] Xu S (2018). A precision therapeutic strategy for hexokinase 1-null, hexokinase 2-positive cancers. Cancer Metab..

[42] Sofer S (2022). A genome-wide CRISPR activation screen reveals Hexokinase 1 as a critical factor in promoting resistance to multi-kinase inhibitors in hepatocellular carcinoma cells. FASEB J..

[43] Floridi A (1981). Effect of lonidamine on the energy metabolism of Ehrlich ascites tumor cells. Cancer Res..

[44] Uhlen M (2015). Tissue-based map of the human proteome. Science.

[45] Sauer T (2022). Protein expression of AEBP1, MCM4, and FABP4 differentiate osteogenic, adipogenic, and mesenchymal stromal stem cells. Int. J. Mol. Sci..

[46] Perez-Riverol Y (2022). The PRIDE database resources in 2022: A Hub for mass spectrometry-based proteomics evidences. Nucleic Acids Res..

[47] UniProt Consortium, T (2018). UniProt: The universal protein knowledgebase. Nucleic Acids Res..

[48] Taylor SC, Berkelman T, Yadav G, Hammond M (2013). A defined methodology for reliable quantification of Western blot data. Mol. Biotechnol..

[49] Dressler FF, Bragelmann J, Reischl M, Perner S (2022). Normics: Proteomic normalization by variance and data-inherent correlation structure. Mol. Cell. Proteomics.

[50] Bramer LM, Irvahn J, Piehowski PD, Rodland KD, Webb-Robertson B-JM (2021). A review of imputation strategies for isobaric labeling-based shotgun proteomics. J. Proteome Res..

[51] Zitnik M, Zupan B (2012). NIMFA: A python library for nonnegative matrix factorization. J. Mach. Learn. Res..

[52] Szklarczyk D (2019). STRING v11: Protein-protein association networks with increased coverage, supporting functional discovery in genome-wide experimental datasets. Nucleic Acids Res..

