# Supplementary figures and images for: Proteomic analyses identify HK1 and ATP5A to be overexpressed in distant metastases of lung adenocarcinomas compared to matched primary tumors
Source: Sci Rep. 2023 Nov 28;13:20948. doi: 10.1038/s41598-023-47767-5 (PMC10684588; doi:10.1038/s41598-023-47767-5)

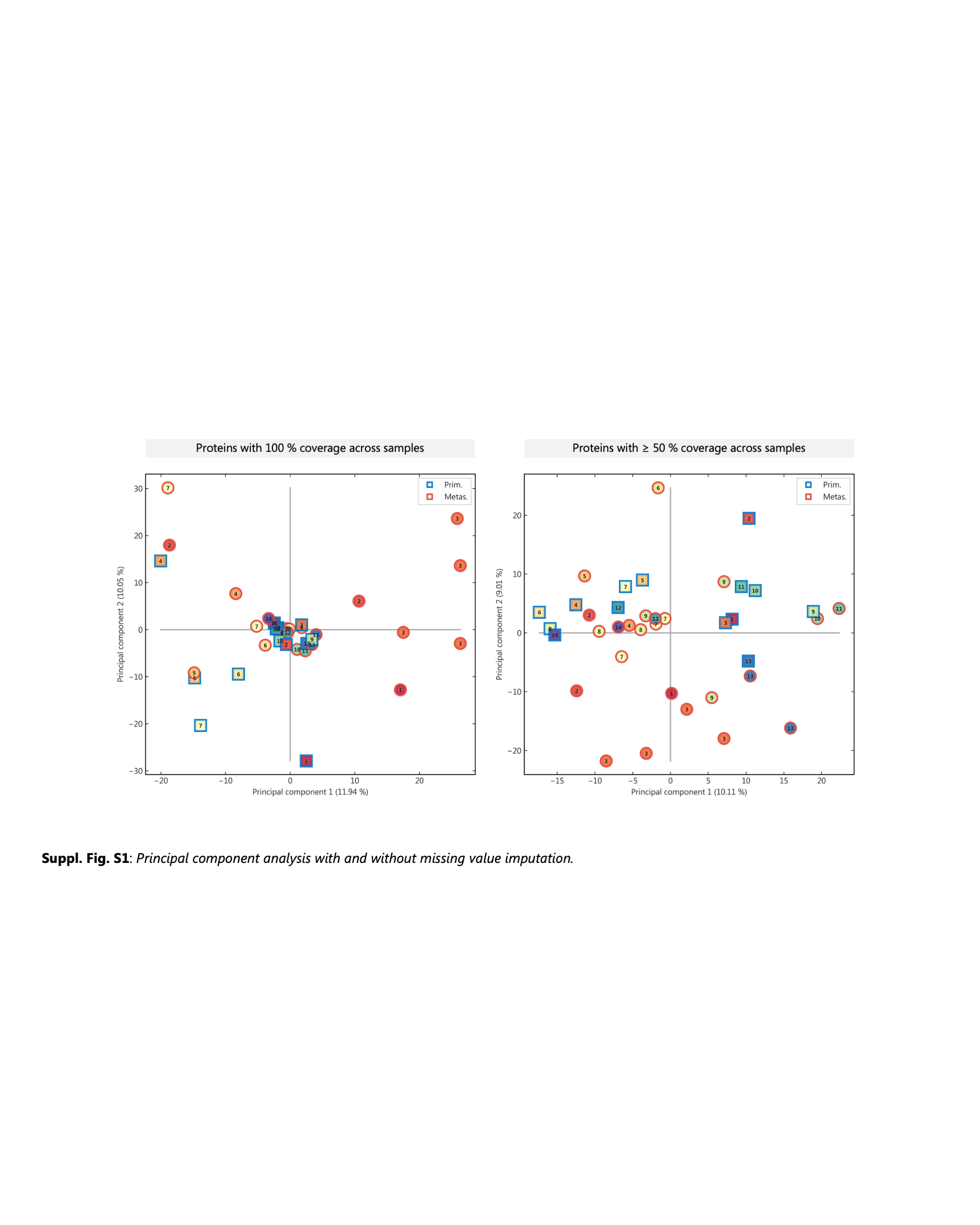

Supplement: Supplementary file 1 — Supplementary Figure S1. [file 41598_2023_47767_MOESM1_ESM.png]

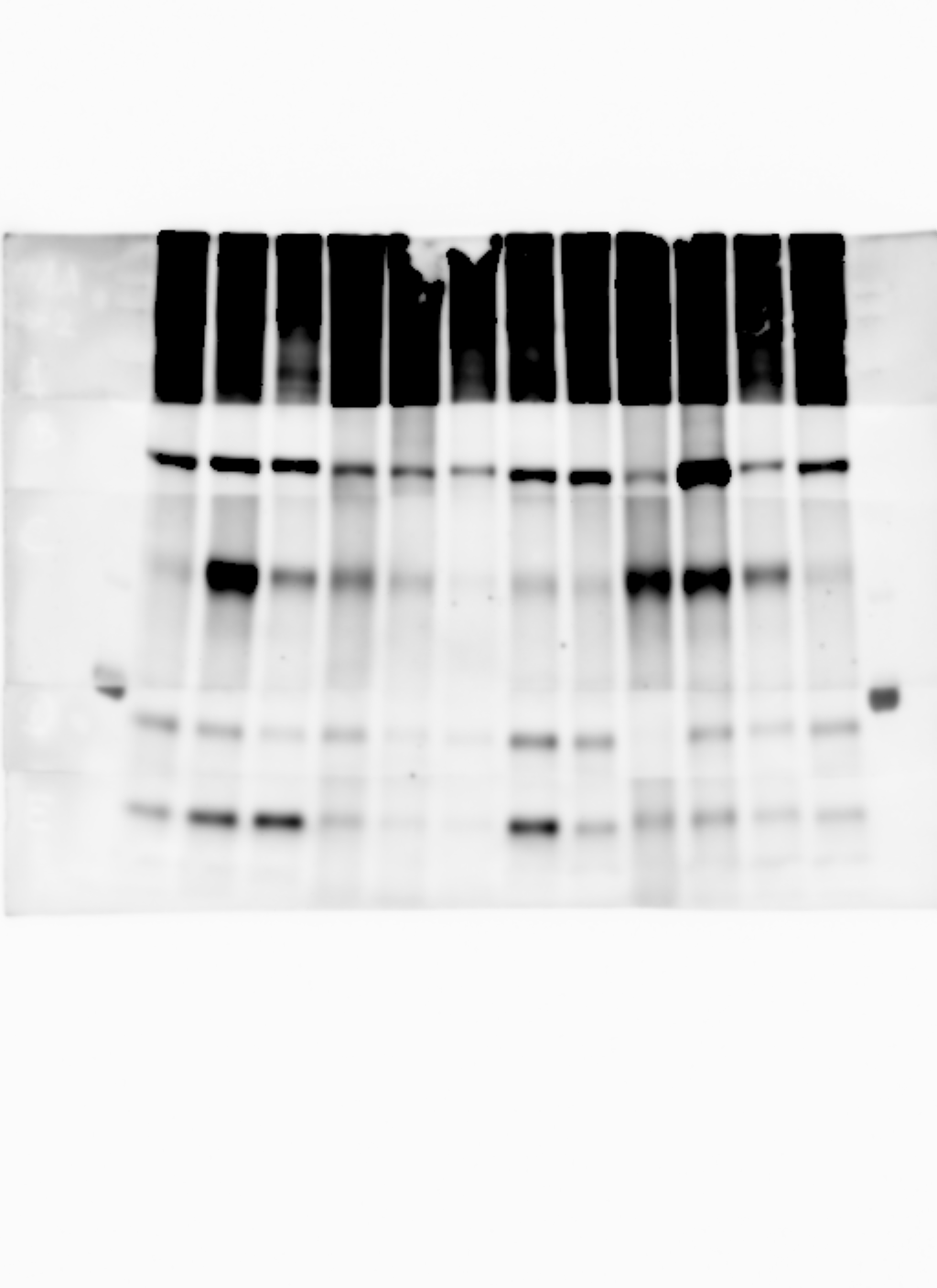

Supplement: Supplementary file 2 — Supplementary Figure S2. [file 41598_2023_47767_MOESM2_ESM.tif]

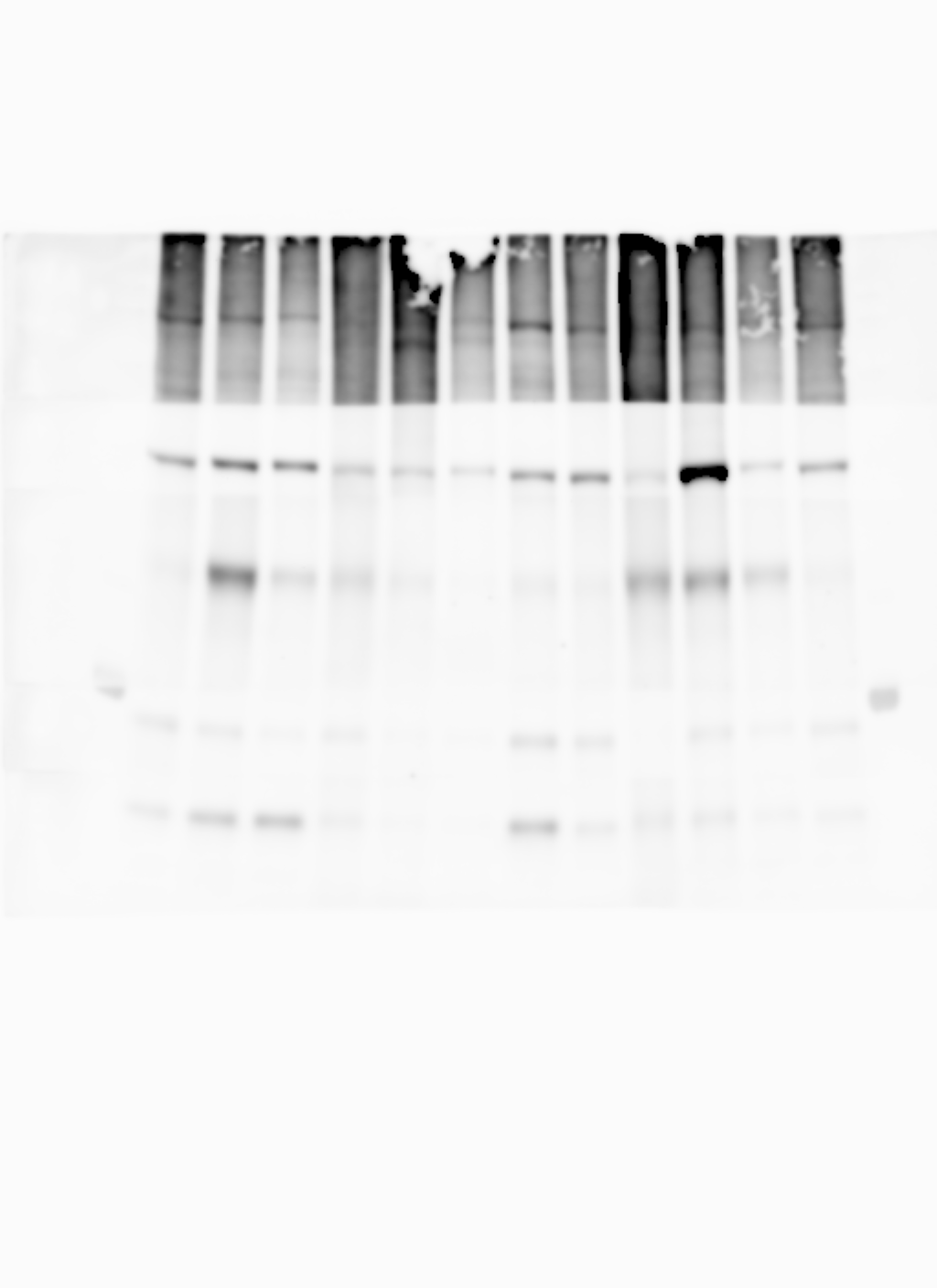

Supplement: Supplementary file 3 — Supplementary Figure S3. [file 41598_2023_47767_MOESM3_ESM.tif]

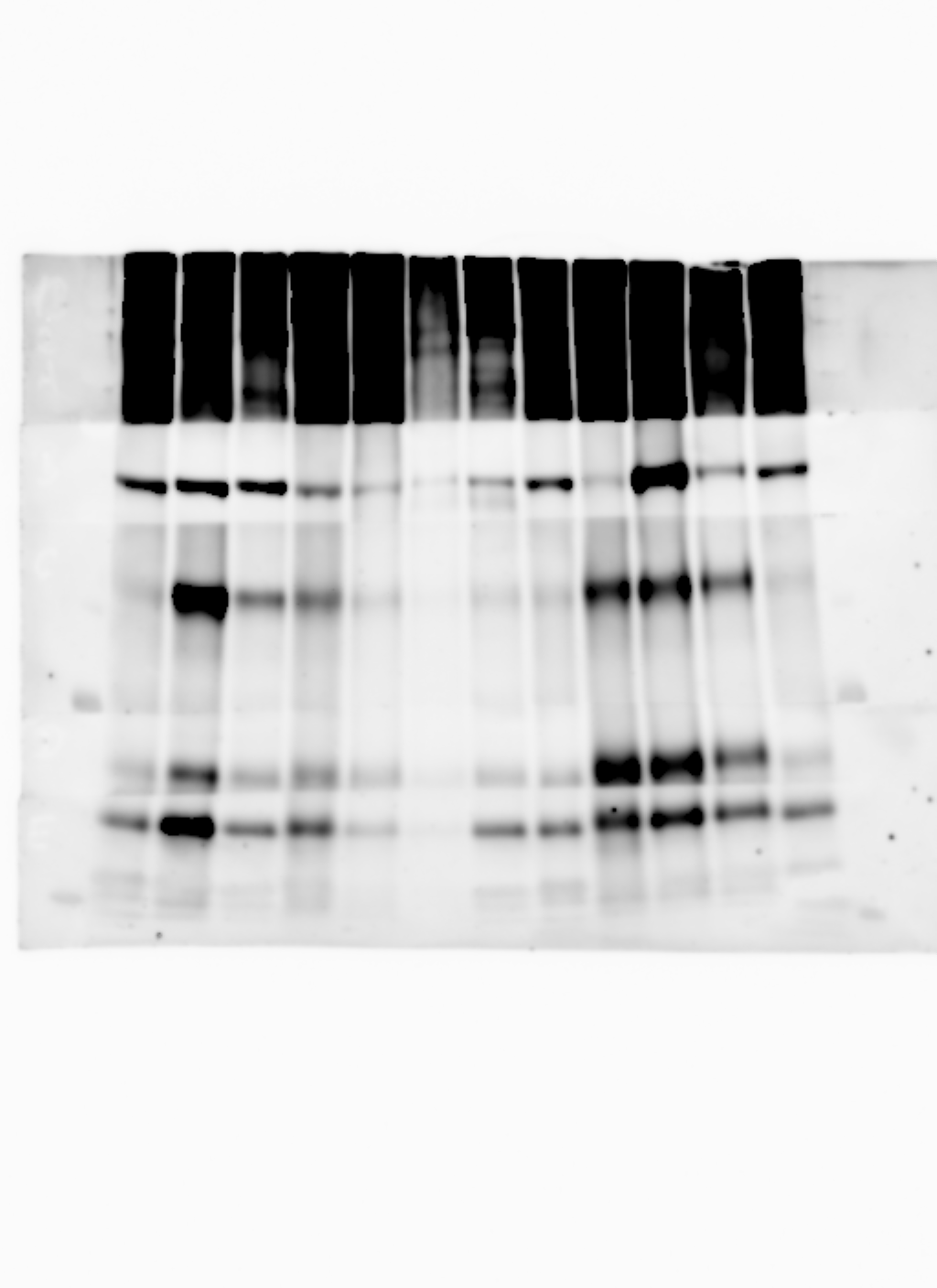

Supplement: Supplementary file 4 — Supplementary Figure S4. [file 41598_2023_47767_MOESM4_ESM.tif]

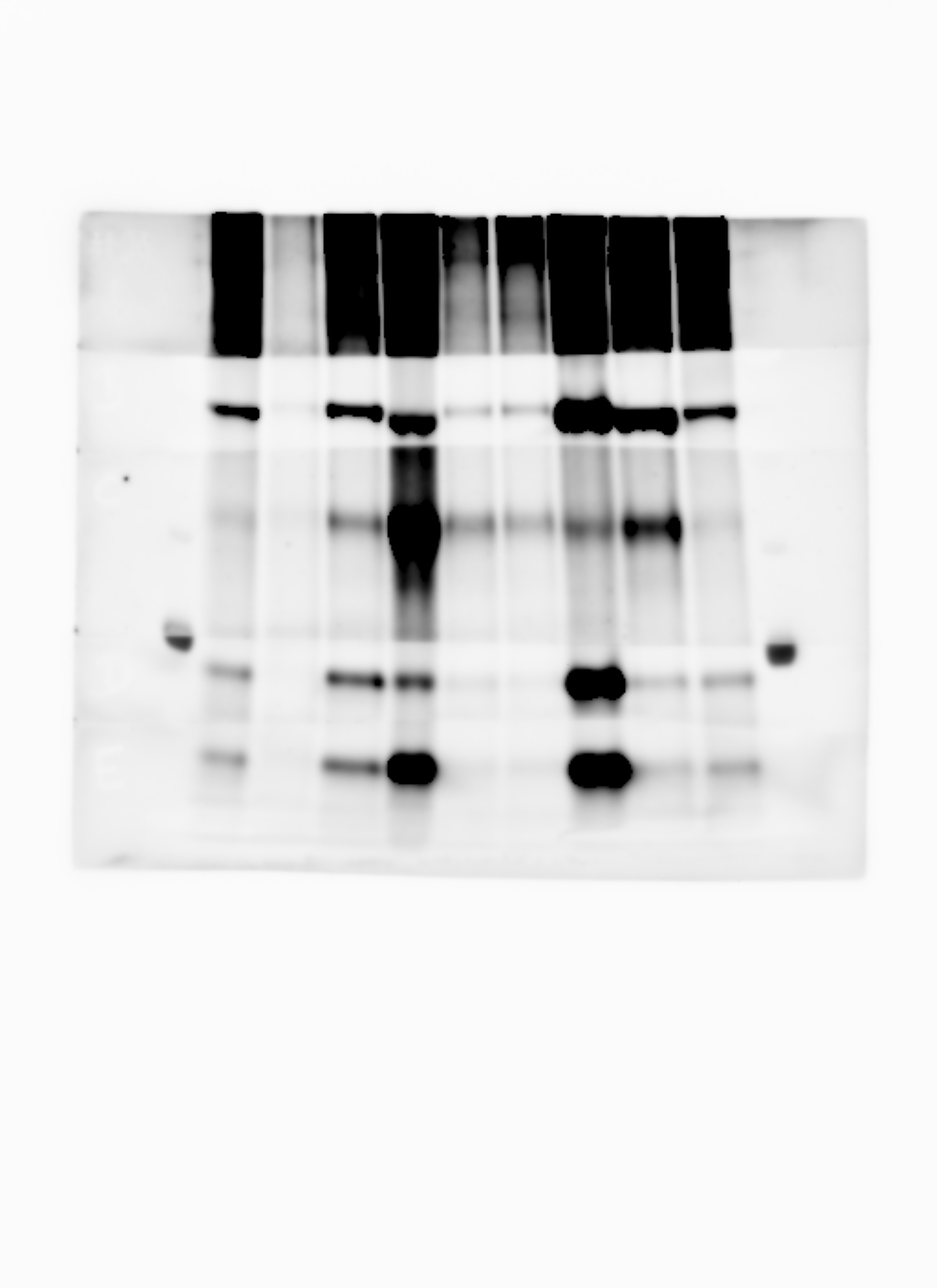

Supplement: Supplementary file 5 — Supplementary Figure S5. [file 41598_2023_47767_MOESM5_ESM.tif]

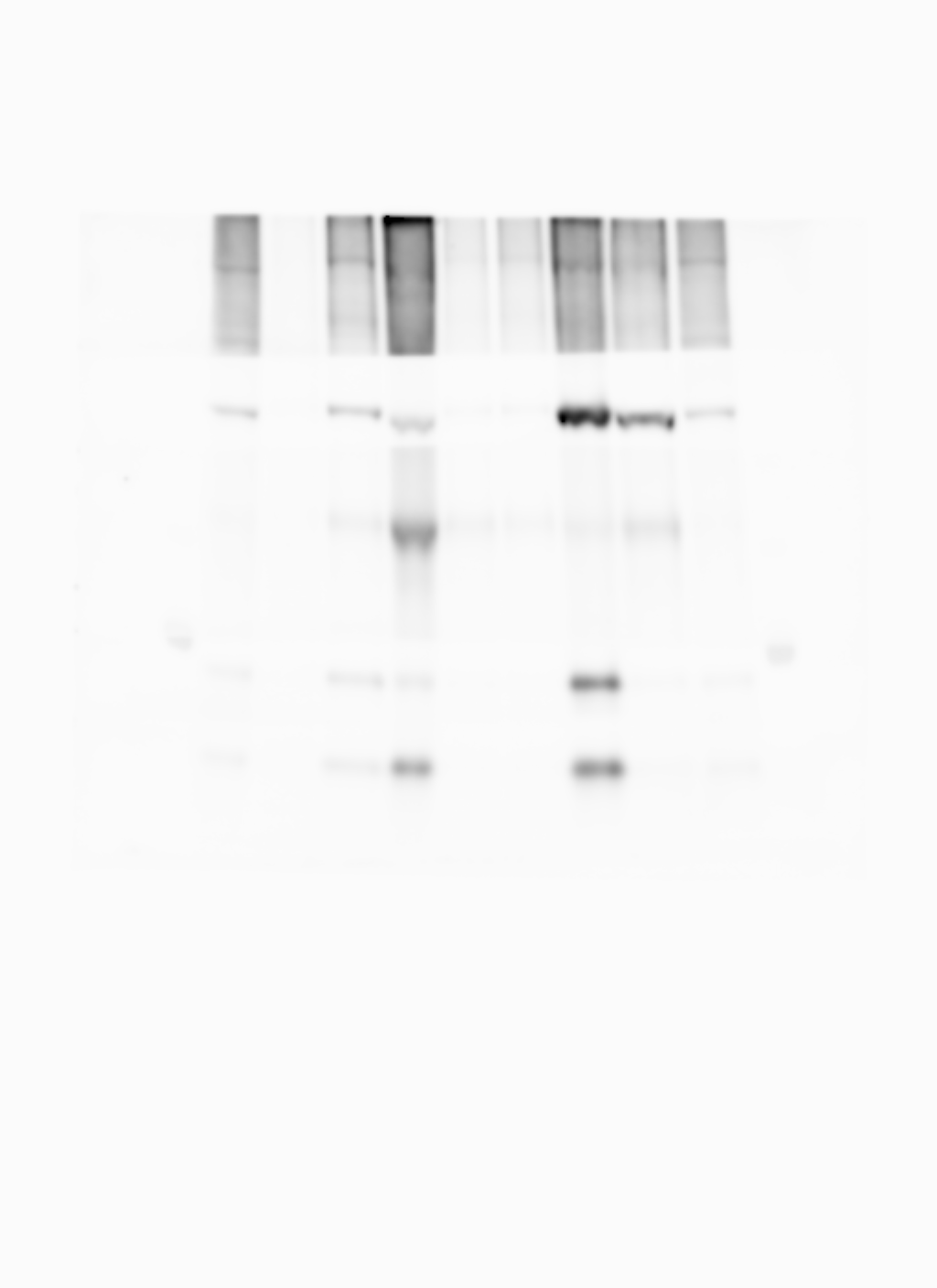

Supplement: Supplementary file 6 — Supplementary Figure S6. [file 41598_2023_47767_MOESM6_ESM.tif]

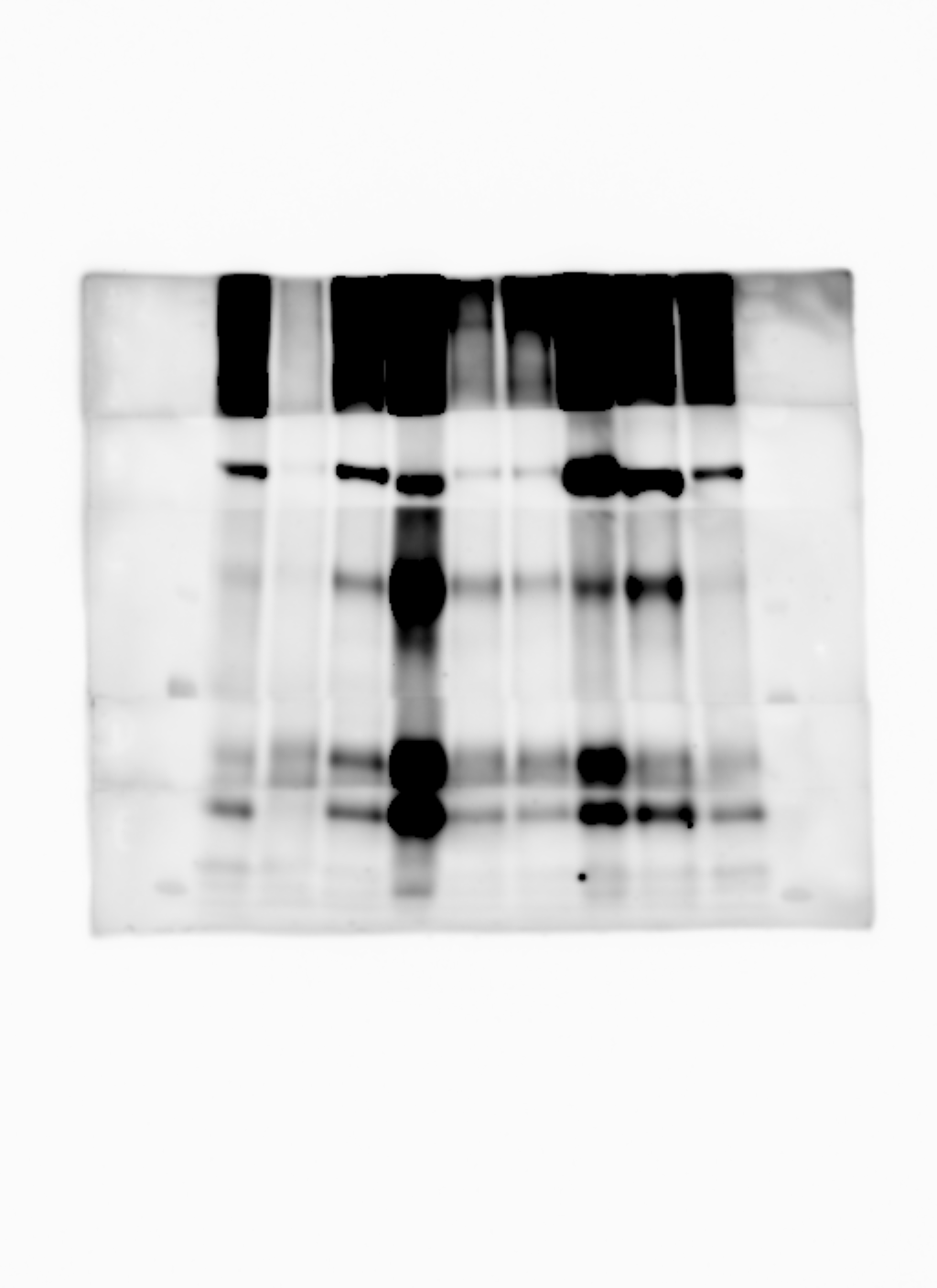

Supplement: Supplementary file 7 — Supplementary Figure S7. [file 41598_2023_47767_MOESM7_ESM.tif]
